# Ultrafast excited-state proton transfer dynamics using linearized pair-density functional theory

**DOI:** 10.1039/d6sc01160h

**Published:** 2026-06-22

**Authors:** Helen S. Clifford, Aniruddha Seal, Laura Gagliardi

**Affiliations:** a Department of Chemistry and Chicago Center for Theoretical Chemistry, University of Chicago Chicago IL 60637 USA; b Pritzker School of Molecular Engineering, University of Chicago Chicago IL 60637 USA lgagliardi@uchicago.edu

## Abstract

Accurate simulation of excited-state bond-rearrangement dynamics remains a major challenge since photoinduced reactions can often involve significant changes in electronic structure along excited-state reaction pathways. Describing such processes requires an electronic structure method that provides balanced descriptions of all electronic states across the nuclear configuration space, while remaining computationally feasible for molecular dynamics. Linearized pair-density functional theory (L-PDFT) provides an efficient multireference framework for excited-state simulations by enabling an accurate multistate treatment of excited-state potential energy surfaces. In this work, we assess the performance of L-PDFT for excited-state bond-rearrangement dynamics using excited-state intramolecular proton transfer (ESIPT) as a stringent benchmark. *Ab initio* molecular dynamics simulations are performed for 10-hydroxybenzo[*h*]quinoline, a prototypical ESIPT system that undergoes ultrafast proton migration following photoexcitation. L-PDFT predicts that ESIPT for the molecule occurs within 16 fs, in close agreement with latest ultrafast time-resolved fluorescence experiments. Trajectory analysis reveals an active role of the proton in driving the ESIPT. These results demonstrate that L-PDFT can describe excited-state photodynamics involving bond rearrangements, highlighting its potential for broader light-driven chemical processes, including excited-state reactivity in photocatalytic transition metal-based systems.

## Abstract 2

Excited-state bond-rearrangement dynamics with L-PDFT yields experimentally consistent mechanisms and timescales.

## Graphical abstract

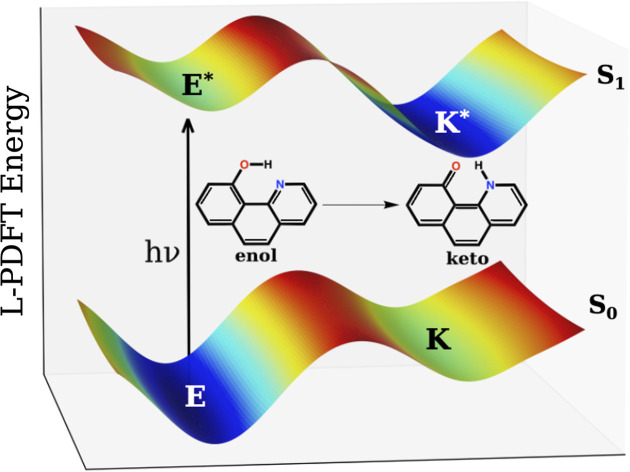

## 1 Introduction

Photoexcitation promotes molecules to excited states, enabling ultrafast rearrangements of the electronic structure and bond-rearrangement pathways that are inaccessible in the ground state. Representative examples of these processes include excited-state proton transfer enabling organic optoelectronic materials,^1,2^ biological imaging,^3,4^ retinal photoisomerization that underlies vision,^5–7^ and photocatalytic reactions initiated by excited state charge-transfer.^8–10^ Additionally, excited-state proton transfer is critical for photoacid chemistry.^11–16^ Such processes involving photoacids are crucial for biomedical therapies^17,18^ as well as in light-driven temporal and spatial pH control for pH-sensitive materials and photoswitches.^18–20^

*Ab initio* molecular dynamics provides a natural route to study such processes in real time, but accurately simulating excited-state bond-rearrangement dynamics places strict demands on the underlying electronic structure method. This requires reliable excited-state energies and analytic nuclear gradients at a reasonable computational cost. Single-reference electronic structure methods are therefore appealing as practical sources of energies and forces. In particular, Kohn–Sham density functional theory (KS-DFT) is widely used because of its computational efficiency and its generally reliable performance for many closed-shell organic and biomolecular systems.^21,22^ For excited states, time-dependent DFT (TD-DFT) offers an inexpensive linear-response framework.^23–25^ However, single-reference methods can become challenging to use when a given wave function is inherently multiconfigurational and characterized by strong electron correlation.^26^ This limitation is especially relevant for photochemical dynamics, where multiconfigurational effects frequently arise due to near-degeneracies, open-shell transition metal complexes with multiple low-lying states, and bond-breaking processes.

Utilizing multireference methods is essential for studying excited-state intramolecular proton transfer (ESIPT) processes with strong static correlation. Although density functional theory has been widely used to study such processes, the proton-transfer coordinate is intrinsically coupled to changes in the electronic structure. Multiconfigurational approaches therefore provide a natural framework for describing the electronic structure consistently throughout the ESIPT.

The complete active space self-consistent field (CASSCF)^27–29^ method provides a means to capture electron (static) correlation by explicitly considering multiple electronic configurations within an active space and can be augmented with post-SCF treatments to recover correlation external to the active space. Common post-CASSCF choices include *n*-electron valence state second-order perturbation theory (NEVPT2)^30^ and CAS second-order perturbation theory (CASPT2),^31^ which can be accurate but are often too expensive for routine excited-state dynamics. Multiconfiguration pair-density functional theory (MC-PDFT)^32^ provides a cost-effective alternative for recovering dynamic correlation by combining a multiconfigurational reference wave function with an on-top density functional. Because it avoids the expensive steps of multireference perturbation theories, MC-PDFT can deliver improved energetics at a fraction of the cost and has been applied successfully across a range of problems, from ground-state reactivity to excited-state photophysics.^33–42^ Despite these successes, MC-PDFT is fundamentally a single-state theory; its state energies are obtained independently. As a result, it can fail to provide accurate excited-state potential energy surfaces, thus limiting its application for excited-state dynamics simulations.

Linearized pair-density functional theory (L-PDFT) is a multistate framework that improves upon these limitations of MC-PDFT.^43^ Through the introduction and diagonalization of an effective Hamiltonian, L-PDFT recovers the correct topology of the potential energy surface around nearly-degenerate states. Moreover, L-PDFT is cheaper than MC-PDFT: its computational cost scales as a constant with the number of states in the state-averaging procedure, whereas MC-PDFT scales linearly with that number.^43,44^ L-PDFT has shown good agreement with multireference benchmarks such as NEVPT2 for vertical excitation energies,^45^ and has also been demonstrated for internal conversion-mediated dynamics without bond breaking.^44,46^ Moreover, Barbatti and co-workers showed that L-PDFT can be more robust than XMS-CASPT2 for on-the-fly dynamics in representative cases, with fewer single-point convergence failures and improved energy conservation along trajectories.^47^

However, it remains important to assess L-PDFT in bond-rearrangement regimes that are central to many photochemical applications, including excited-state cross-coupling reactions in transition metal-based photocatalysis,^9,48,49^ photoinduced proton-coupled electron transfer,^50–52^ solar energy storage and conversion processes through reversible switching of molecular photoswitches^53–55^ and light-responsive drug delivery.^56,57^

**Fig. 1:**
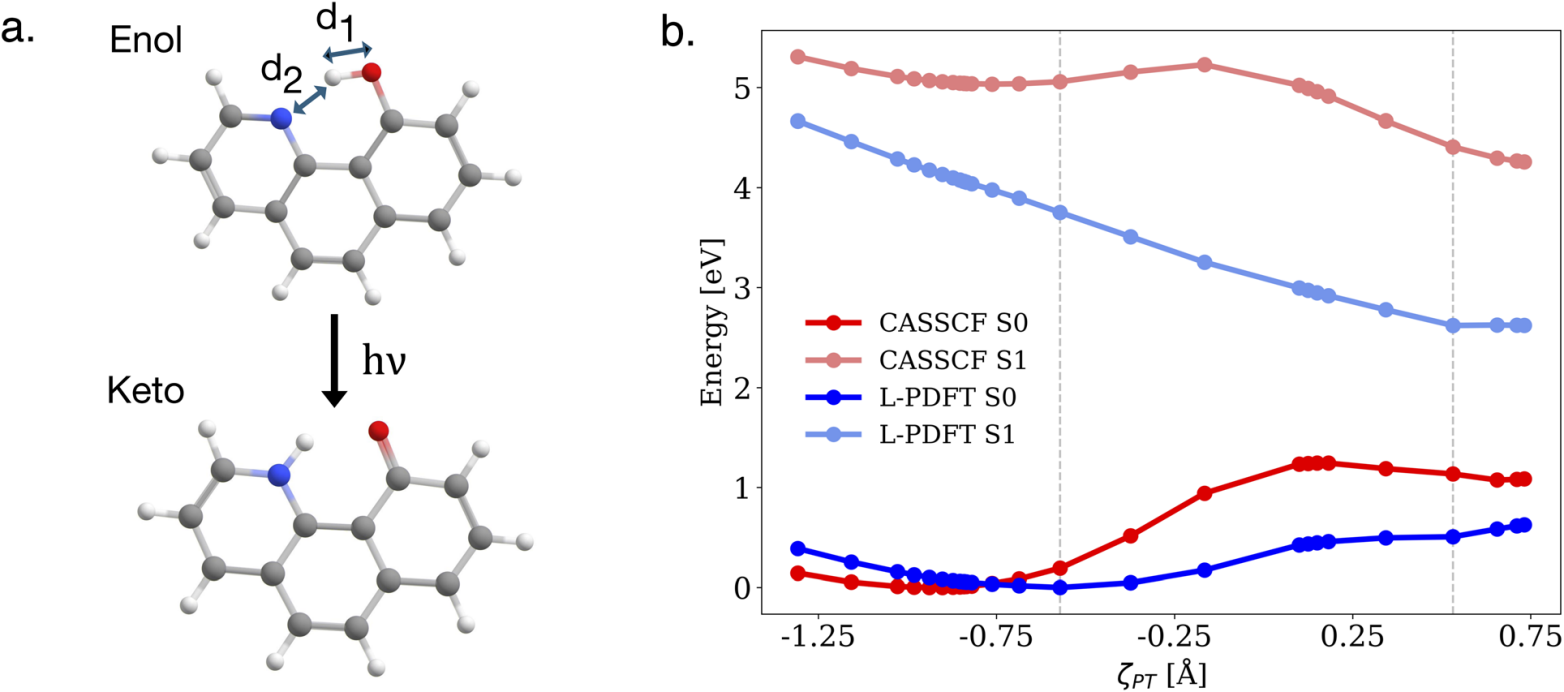
(a) Enol and keto tautomer structures of 10-hydroxybenzo[*h*]quinoline (HBQ) involved in intramolecular excited-state intramolecular proton transfer (ESIPT); atom colors: C (gray), N (blue), O (red), and H (white). (b) Ground-state (S_0_) and first excited singlet-state (S_1_) potential-energy profiles along the ESIPT coordinate, *ζ*_PT_ = *d*_1_ − *d*_2_, computed with SA(2)-CAS(4,4) and L-PDFT (with the tPBE on-top functional) using the 6-31G** basis set. Geometries were optimized at the corresponding level of theory, and energies are referenced to the S_0_ minimum for each method. Vertical dotted gray lines mark the *ζ*_PT_ values of the equilibrium enol and keto structures. The leftmost vertical line corresponds to the enol form which is a minimum on L-PDFT S_0_ and the rightmost vertical line corresponds to the keto form which is a minimum on L-PDFT S_1_.

Accordingly, establishing the robustness of L-PDFT in bond-rearrangement regimes is a necessary step toward predictive photodynamics simulations. Here, we evaluate L-PDFT for excited-state bond-rearrangement dynamics, using the ESIPT in 10-hydroxybenzo[*h*]quinoline (HBQ) as a stringent case study. HBQ is a prototypical ESIPT chromophore whose ultrafast enol-to-keto tautomerization has been extensively characterized (Fig. 1a). We choose to study HBQ here for three reasons: (i) the electronic reorganization on S_1_ along the proton-transfer coordinate gives rise to significant multiconfigurational character (Section SV), making it a meaningful test of L-PDFT's multistate description; (ii) HBQ is among the most thoroughly characterized ultrafast photochemical systems, providing high-quality experimental benchmarks^58,59^ and (iii) existing TD-DFT ESIPT timescales are longer than experimental timescales,^58,60^ suggesting that a multireference treatment may be advantageous.

ESIPT in HBQ involves the transfer of the phenolic proton from the enol tautomer to the ring nitrogen, forming the keto tautomer. The two forms are separated by a substantial barrier in the electronic ground state. Upon photoexcitation, however, the ESIPT reaction becomes thermodynamically favorable on the excited-state surface, enabling rapid proton transfer. We use this reaction as a compact test case to assess whether L-PDFT can provide reliable excited-state potential energy surfaces and analytic nuclear forces for bond-rearrangement dynamics.

We first compute the ground (S_0_) and first excited singlet (S_1_) states of HBQ with L-PDFT and map the corresponding potential energy profiles along the proton-transfer coordinate. Following that, we perform *ab initio* excited-state dynamics on S_1_ to simulate the ultrafast ESIPT event. From these trajectories, we extract the proton transfer timescale, characterize the role of the proton in the proton-transfer, and investigate the structural evolution of the molecule along the reaction. The resulting proton-transfer timescale of ∼16 fs is in close agreement with latest experiments of HBQ. Collectively, these results provide the first demonstration of excited-state bond-rearrangement dynamics with L-PDFT and establish its utility for simulating photochemical processes that involve bond rearrangements within an affordable multireference framework. Furthermore, this work opens the door to applications of L-PDFT to larger photoactive systems and photocatalytic transition metal complexes.

## 2 Computational methods

We briefly recall the essentials of L-PDFT.^43^ In L-PDFT, one introduces an effective Hamiltonian that is a functional of the state-averaged density and on-top pair density, whose eigenvalues are linear approximations to the corresponding MC-PDFT state energies. Concretely, the L-PDFT energy for state |Γ〉 is obtained as the first-order Taylor expansion of the MC-PDFT energy about a chosen zero-order density, *γ̌*.^32,43^1
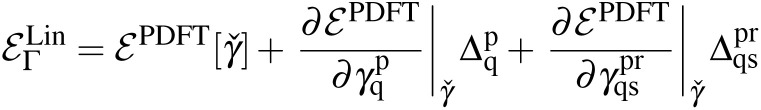
where *γ*^p^_q_ and *γ*^pr^_qs_ denote the one- and two-particle reduced density matrix (1-RDM and 2-RDM) elements for state |Γ〉, respectively. The quantities *γ̌*^p^_q_ and *γ̌*^pr^_qs_ are the corresponding elements of the zero-order density, and Δ^p^_q_ = *γ*^p^_q_ − *γ̌*^p^_q_ and Δ^pr^_qs_ = *γ*^pr^_qs_ − *γ̌*^pr^_qs_ are the deviations from the zero-order values. The zero-order MC-PDFT energy appearing in eqn (1) is:^32^2
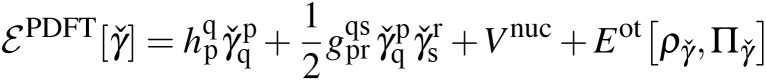
where *h*^q^_p_ and *g*^qs^_pr_ are the one- and two-electron integrals, *V*^nuc^ is the nuclear-repulsion energy, and *E*^ot^[*ρ*_*γ̌*_, Π_*γ̌*_] is a functional of the zero-order electron density (*ρ*_*γ̌*_), the on-top pair density (Π_*γ̌*_), and their derivatives. The zero-order densities are taken to be the weighted average densities used in the state-averaged regime.

This construction leads to the L-PDFT effective Hamiltonian in the state-averaged (SA)-CASSCF model space. Because the energies are obtained by direct diagonalization, L-PDFT provides a multistate description without requiring an iterative procedure to determine an intermediate basis for an effective model-space Hamiltonian^67^ or the solution of a set of linear perturbation-theory equations,^68^ and it does not encounter the intruder-state problem that can arise in perturbative treatments.^69^

We describe ESIPT in HBQ using a four-electron, four-orbital (4e,4o) active space (shown in Fig. S1) and employ a two-state averaged reference over the lowest two singlet states. All electronic-structure calculations were performed with PySCF^70,71^ using PySCF-Forge,^72^ with spin-adapted configuration state functions and no spatial symmetry enforced. All calculations employ equal weights in the state-averaging to provide a balanced orbital description of both the ground and the first excited singlet state. All calculations used the 6-31G** basis set,^61–63^ and the tPBE on-top functional^32,64^ was utilized for all L-PDFT calculations. To validate this choice, we computed the potential energy profiles along the proton-transfer coordinate using the def2-TZVP basis set^65^ and found that the qualitative features are preserved (Section SVI), indicating that 6-31G** provides a reliable description at a reduced computational cost. Additionally, L-PDFT is also known to be less sensitive to the basis set size than NEVPT2 and other post-SCF methods.^43,45,73,74^

*Ab initio* molecular dynamics were performed using an ASE–PySCF interface,^75,76^ propagating nuclei with the velocity-Verlet integrator using a 0.5 fs timestep, with analytic gradients evaluated at the L-PDFT level of the theory. Additional computational details are provided in Section SI of the SI.

## 3 Results and discussion

We first compute the vertical excitation energy to the lowest energy singlet excited state of both the enol and keto tautomers, which corresponds primarily to a π → π* transition. Table 1 summarizes the resulting vertical excitation energies from SA-CASSCF and L-PDFT and includes, for comparison, values from TD-DFT,^58,66^ the resolution-of-the-identity coupled-cluster singles and doubles approach (RI-CC2),^58^ and available experimental gas-phase excitation energies.^58^ L-PDFT reproduces the expected ordering of the enol and keto vertical excitation energies with respect to TD-DFT, RI-CC2, and experimental values. In addition, the L-PDFT excitation energies fall within 0.5 eV of the experimental values. In contrast, SA-CASSCF systematically overestimates the excitation energies. L-PDFT improves upon SA-CASSCF, consistent with prior vertical excitation benchmark assessments of L-PDFT.^45^ Next, we compute potential energy scans for the S_0_ and S_1_ states along the proton-transfer coordinate *ζ*_PT_,^60,77–80^ constructed from L-PDFT-optimized geometries (optimization details are provided in Section SI of the SI). The coordinate *ζ*_PT_ is defined as

3 *ζ*_PT_ = *d*(O–H) − *d*(N–H)

**Table 1:** Vertical excitation energies (eV) to the lowest ππ* singlet excited state (S_1_) of the HBQ enol and keto tautomers. For SA-CASSCF and L-PDFT, vertical excitations were evaluated at S_0_ equilibrium geometries for enol and S_1_ for keto, optimized with the corresponding method. TD-DFT and RI-CC2 values, as well as available gas-phase experimental excitation energies, are taken from the literature

Method	Basis	Functional	Active space	Enol	Keto
Exp (from ref. 58)				3.26	1.98
L-PDFT	6-31G**^61–63^	tPBE^32,64^	(4e,4o)	3.66	1.63
SA-CASSCF	6-31G**^61–63^	tPBE^32,64^	(4e,4o)	4.97	2.38
L-PDFT	def2-TZVP^65^	tPBE0	(4e,4o)	3.91	1.88
SA-CASSCF	def2-TZVP^65^	tPBE0	(4e,4o)	4.83	2.49
RI-CC2 (from ref. 58)	SVP			3.64	1.71
TD-DFT (from ref. 58)	SVP	B3LYP		3.35	1.97
TD-DFT (from ref. 66)	def2-TZVP	B3LYP		3.41	2.84
TD-DFT (from ref. 66)	def2-TZVP	PBE0		3.53	2.19

As is apparent in eqn (3), a negative *ζ*_PT_ corresponds to the enol tautomer and a positive *ζ*_PT_ corresponds to the keto tautomer.

As shown in Fig. 1b, the potential energy scan using both L-PDFT and SA-CASSCF captures the expected behavior of ESIPT for HBQ. An increasing *ζ*_PT_ implies progress from the enol to the keto tautomer. Thus, for the ground state S_0_, as *ζ*_PT_ increases there is a rise in energy. This correlates with an uphill and therefore disfavored proton transfer. In contrast, for the excited state S_1_, the energy decreases as the system moves from enol to keto, demonstrating barrierless proton transfer following photoexcitation. This qualitative behavior agrees with prior theory and experimental evidence that ESIPT occurs in the excited state.^58,60,81,82^ Comparison of the L-PDFT and SA-CASSCF scans highlight the impact of dynamic correlation: for both S_0_ and S_1_, the L-PDFT potential energy curves are stabilized relative to SA-CASSCF since L-PDFT incorporates correlation external to the active space. Additionally, the second excited state (S_2_) was well-separated from S_1_ by 0.3–0.79 eV throughout the ESIPT (Fig. S12) allowing for its omission. Previous studies have also noted that the proton transfer occurs entirely in the S_1_ excited state.^80,83^ Further discussion of potential energy surfaces obtained with L-PDFT, including comparisons to other multistate PDFT and perturbation theories, has been presented by Hennefarth *et al.*^43^

For certain molecular systems, excited states involved in bond rearrangements can exhibit multiconfigurational character.^84^ To quantify it along the ESIPT pathway, we compute the M diagnostic,^85^ which measures the extent of static correlation within a system based on the natural orbital occupation number. There exists various multireference metrics based on occupation numbers. We make note of a study by Fogueri *et al.*^86^ that demonstrates a comprehensive study of various multireference diagnostics. It is shown that the M diagnostic has consistent performance relative to other diagnostic metrics based on natural orbital occupation numbers.

Calculation of the M diagnostic along the energetically favorable pathway on S_1_ shows values indicating significant multiconfigurational character for the first excited state of HBQ (Section SV). This underscores the strongly multiconfigurational nature of HBQ undergoing ESIPT and can highlight the utility of modeling excited-state bond-rearrangement dynamics with multireference methods such as L-PDFT. In addition, we analyze the CI expansion of the excited state wave function to characterize the dominant determinants involved in the excited state during the ESIPT. The excited state primarily involves ππ* character^58^ with no single dominant configuration. Further details and M diagnostic values for the full profile can be found in Section SV.

Having established the reliability of the underlying potential energy surfaces, we now turn to the excited-state dynamics. We begin by performing a 1 ps equilibrium *ab initio* dynamics of HBQ in the S_0_ enol basin, generating an ensemble of 10 000 enol configurations from which initial conditions were selected. Because ESIPT on S_1_ is effectively barrierless, mechanistic observables can strongly depend on the initial conditions.^87^ Therefore, it is essential to sample a diverse set of initial geometries to obtain robust ensemble-averaged dynamics. To this end, we featurized each configuration using smooth overlap of atomic position (SOAP) descriptors,^88^ which provide a compact, rotation- and permutation-invariant representation of local atomic environments. We then employ *k*-means clustering, which partitions the set of enol geometries by assigning each geometry to the nearest centroid so as to minimize within-cluster variance (Fig. S4). Since each cluster correlates with a distinct region of the configuration space, we selected the geometry closest to each centroid as the representative geometry of that region. This procedure yields 100 maximally diverse enol geometries that collectively span the equilibrium S_0_ configurational distribution relevant to proton-transfer dynamics (Fig. S4).

The S_0_ enol geometries were then promoted to S_1_*via* a Franck–Condon excitation and used as an initial condition to simulate the ESIPT. Each geometry was used to initiate a 75 fs adiabatic trajectory propagated on the S_1_ surface in the microcanonical ensemble using L-PDFT analytic gradients, since the S_1_–S_0_ gap remains large along the transfer, consistent with previous simulations.^60^ To ensure active space consistency, we monitored total energy conservation along each trajectory and retained only those that remained stable (shown in Fig. S2 and S3). For trajectories that did not conserve the total energy, the HBQ molecule demonstrated significant distortion and loss of planarity of the molecular backbone. Applying this criterion reduced the ensemble from 100 to 72 trajectories, which were used for all subsequent analyses.

**Fig. 2:**
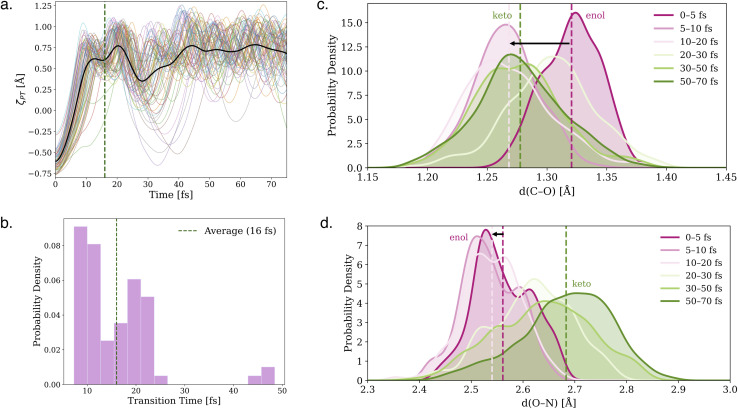
(a) Time evolution of the ESIPT in HBQ along the proton-transfer coordinate *ζ*_PT_ on the S_1_ surface. The ensemble-averaged trajectory is shown in black. The green vertical line marks the mean completion time, defined as the first passage into the keto basin where the proton transfer is considered complete. (b) Distribution of all 72 trajectory transition times (defined as the time at which each trajectory achieves *ζ*_PT_ = 0.79 Å, indicative of proton transfer completion). The vertical green marker denotes the average time each trajectory is deemed complete. Excited-state structural evolution along the S_1_ trajectories: (c) time-resolved probability density of the C–O bond length during the excited-state dynamics. Kernel density estimates (KDEs) of *d*(C–O) are shown for successive time blocks (0–5, 5–10, 10–20, 20–30, 30–50, and 50–70 fs), with each curve normalized to the unit area. Curves are colored by time-block order. Dashed vertical lines mark the mean *d*(C–O) in the time blocks 0–5 fs which marks the “enol” form, 10–20 fs which marks the “nascent keto form” and the final 50–70 fs block which marks the “relaxed keto form” of HBQ; (d) same as panel (c), but for the O–N distance, *d*(O–N), plotted as time-resolved KDEs. Dashed vertical lines denote the mean *d*(O–N) in the first and last time blocks. The respective black arrows on each of the plots highlight the net shift in the distribution over the average reaction time of 16 fs.

Fig. 2a displays the time evolution of the proton transfer coordinate *ζ*_PT_, along the S_1_ trajectories. To quantify the ESIPT timescale, we define the proton-transfer time for each trajectory as the time at which *ζ*_PT_ reaches the value characteristic of the optimized excited-state keto minimum (*ζ*_PT_ = 0.79 Å), indicating the completion of the proton transfer. The HBQ ESIPT timescale is then taken as the average of these completion times over the trajectory ensemble. We find that the proton transfer occurs within 16 ± 8 fs. This value agrees closely with the latest time-resolved fluorescence experiments reporting a timescale of 12 ± 6 fs.^59,89^ Our result of a 16 fs timescale is notably faster than early transient-absorption measurements by Schriever *et al.*, which, limited by a 30 fs time resolution, inferred a 30–40 fs transfer time.^58^ Our L-PDFT ESIPT timescale is also shorter than TD-DFT based ESIPT timescales of 30–50 fs.^58,60^ A 29 fs ESIPT timescale is reported by a molecular dynamics simulation of HBQ using electronically embedded multiconfigurational Shepard interpolation.^90^ An excited-state molecular dynamics study of HBQ using ADC(2) was also performed using the JADE dynamics package and found an estimated ESIPT timescale of 20 fs.^91^ Furthermore, computational time-resolved X-ray studies report a 12–20 fs timescale for HBQ ESIPT.^80,83^ Existing experimental and theoretical studies consistently characterized ESIPT in HBQ as an ultrafast process occurring on a timescale of a few tens of femtoseconds.^58,60,80,83,91–93^ The L-PDFT timescale reported here falls within this range. In all, our results suggest that L-PDFT captures the ultrafast character of the HBQ ESIPT process and provides a promising foundation for future methodological developments.

This is further backed up by Fig. 2b that illustrates the normalized distribution histogram of all 72 trajectory transition times which is defined as the time at which each trajectory achieves *ζ*_PT_ = 0.79 Å, indicative of proton transfer completion. This plot shows the spread of HBQ ESIPT transition times and makes evident again that a majority of our calculated trajectories achieved completion of ESIPT earlier than what is predicted by TD-DFT.^58,60^

To gain insights into the reaction mechanism, we examine the structural evolution of HBQ during the ESIPT process. Specifically, the carbonyl (C)–O and O–N distances were observed as a function of time during the proton transfer. By virtue of the ESIPT, the enol isomer tautomerizes to the keto isomer, causing the hydroxyl-group oxygen to form a double bond to its bonded carbon. Characteristically, forming the carbonyl of the keto isomer causes the C–O bond distance to shorten. This behavior can be seen in Fig. 2c, in agreement with Raucci.^60^

Next, we display the O–N distance against reaction progress time to understand if the skeletal structure of HBQ facilitates the proton transfer between O and N. Fig. 2d shows the time-resolved probability density for O–N distance throughout the dynamics. Our results show that during ESIPT of HBQ, the O and N maintain a relatively constant distance throughout the portion of the predicted ESIPT timescale (16 fs). Subsequently, the system relaxes once it reaches the keto structure as is evidenced by the shifting of the later time blocks. Additionally, changes in C–C distances within the HBQ molecular backbone were investigated and yielded small fluctuations (Fig. S8), indicating that significant structural changes are around the proton transfer site rather than being spread about the skeletal backbone of HBQ.

Following the prescription in Schriever *et al.*,^58^ we investigate the role of the proton during the ESIPT. The role of the proton includes passive, active, and semi-passive roles. The proton is defined as passive when its migration is entirely governed by the contraction of O–N. In an active proton role, the stretching of the O–H bond drives the ESIPT without the O–N bond shortening. Lastly, in a semi-passive proton role, skeletal deformations of the HBQ structure initially trigger proton transfer to allow stretching of the O–H bond. Lee *et al.* performed ultrafast time-resolved fluorescence measurements on HBQ and its deuterated analogue (DBQ) and reported an H/D isotope dependence consistent with ballistic proton migration, implying that motion along the proton transfer coordinate is initiated rather than being driven by prior heavy-atom rearrangement (active role).^59^

**Fig. 3:**
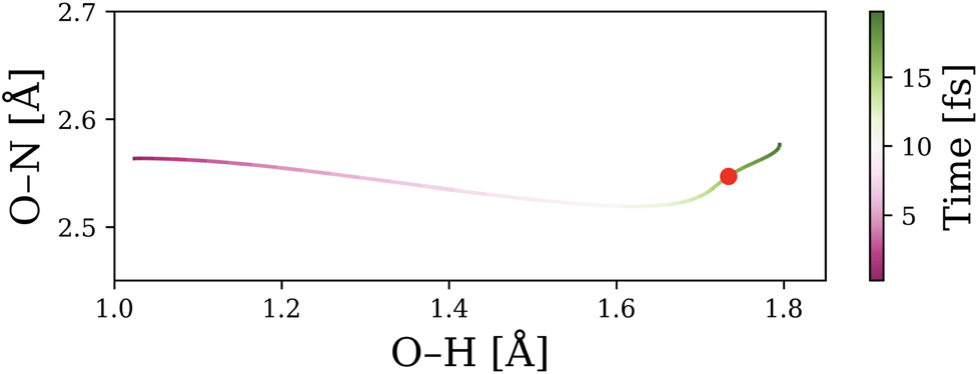
Averaged S_1_ ESIPT trajectory of HBQ projected onto the O–H and O–N distances to assess the active/passive role of the transferring proton. The mean trajectory is colored according to time (color bar), and the red marker denotes the point at which the averaged trajectory reaches the ESIPT completion time of 16 fs. The full time evolution of the averaged trajectory is shown in Fig. S7.

To explain this mechanistic picture that emerges from the L-PDFT simulations, we analyze the ensemble-averaged time evolution of the O–H and O–N distances. As shown in Fig. 3, the O–N distance remains nearly constant while the O–H bond elongates rapidly during proton transfer, indicating that proton transfer proceeds predominantly from the O–H stretch with minimal concomitant skeletal deformation. This is further supported by the strong correlation between *ζ*_PT_ and the O–H distance (Fig. S6), demonstrating that reaction progress is tightly coupled to O–H elongation.^59^ Moreover, examination of the full trajectories is indicative of structural relaxation of the molecular backbone observed following proton transfer (Fig. S7).

The available experimental and theoretical studies are consistent with this picture, indicating that HBQ undergoes an ultrafast intramolecular ESIPT with weak solvent dependence.^90,92–95^ In particular, Higashi and Saito found comparable ESIPT for HBQ in the gas phase and in solution, supporting the use of gas-phase dynamics as a reasonable model for the intrinsic proton-transfer timescale of HBQ.^90^ However, we emphasize that this behavior should not be interpreted as a universal feature of ESIPT. Solvent interactions can affect proton-transfer dynamics in other chromophores, and inclusion of solvent effects may therefore be necessary to obtain quantitative agreement with experiment.^96^

Following the approach of Barbatti and co-workers,^97,98^ we have also computed time-resolved fluorescence spectra using a constant transition dipole moment (Fig. S17), to have a direct comparison with experimental observables. This analysis yields a fluorescence decay time constant of 6–10 fs, compared with the experimental value of 12 fs.^59^ This approximate treatment provides a reasonable description. Analytic transition dipole moments for L-PDFT are currently under development and will further improve quantitative agreement with experiment.

## 4 Conclusions

In this work, we demonstrate the capability of L-PDFT for excited-state bond-rearrangement dynamics, using excited-state intramolecular proton transfer in HBQ as a stringent benchmark. L-PDFT reproduces the vertical excitation energies at the enol and keto structures in good agreement with experiment,^58^ and provides a physically consistent description of the excited-state potential energy surface along the proton-transfer coordinate while capturing the multireference character, quantified with the M diagnostic.

Because HBQ ESIPT proceeds downhill on S_1_, the proton transfer time depends on the distribution of initial Franck–Condon geometries. Hence, we initialize an ensemble of *ab initio* S_1_ trajectories from a diverse, representative set of Franck–Condon configurations selected *via* SOAP-based clustering, and predict an ultrafast ESIPT timescale of 16 fs. This value is in close agreement with the latest ultrafast time-resolved fluorescence measurements (12 ± 6) fs.^59,89^ Beyond the timescale, L-PDFT reproduces key mechanistic signatures observed experimentally. Analysis of the structural evolution supports an active role of the proton; the O–H elongation in HBQ drives the reaction while the O–N distance remains nearly constant, consistent with the picture inferred from isotope-dependent fluorescence experiments.^59^ We note, however, that excited-state dynamics can be sensitive to the choice of initial conditions.^99–102^ In future work, analytic L-PDFT Hessians, which are under development, will enable Wigner sampling based on L-PDFT vibrational frequencies and allow us to systematically assess the sensitivity of the predicted ESIPT dynamics to the initial ensemble. Together, these results provide the first demonstration of excited-state proton transfer dynamics with L-PDFT and establish it as a multireference approach for simulating photochemical bond rearrangements in real time.

Looking ahead, an important direction is to extend L-PDFT dynamics to treat intersystem crossing events by incorporating spin–orbit coupling,^103^ enabling non-adiabatic simulations of photocatalytic reaction dynamics in transition-metal complexes where spin-state changes are integral to reactivity.^9^ In parallel, L-PDFT opens a pathway to the high-quality multireference excited-state data and can be naturally combined with our recently proposed weighted active space protocol (WASP)^37^ to enable multireference machine-learned potentials for excited-state dynamics. Coupling L-PDFT with WASP provides a practical workflow for producing consistent excited-state energies and forces across bond rearrangements and regions of changing electronic character, while maintaining stable active-space representations along nonequilibrium geometries. Beyond energies and forces, the development of L-PDFT transition dipole moments would further expand its applicability by enabling quantitative accuracy in absorption and emission spectra, where spectral intensities depend on the square of the transition dipole moment. Together, these developments will position L-PDFT for predictive excited-state dynamics in transition-metal photocatalysis, where dense manifolds of electronic states underpin light-driven bond activation.

## Author contributions

Helen S. Clifford: conceptualization; methodology; investigation; formal analysis; writing – original draft; writing – review and editing. Aniruddha Seal: conceptualization; methodology; investigation; formal analysis; writing – original draft; writing – review and editing. Laura Gagliardi: conceptualization; funding acquisition; resources; supervision; writing – review and editing.

## Conflicts of interest

There are no conflicts to declare.

## Supplementary Material

SC-017-D6SC01160H-s001

## Data Availability

The data supporting this article have been included as part of the supplementary information (SI). Supplementary information: discussion of electronic structure calculations and methods used, active space of HBQ, active space stability of S_0_ and S_1_, selection of initial conditions, discussion of O–H *vs. ζ*_PT_, determination of the proton role, discussion of the M diagnostic, L-PDFT potential energy surfaces, energy conservation of trajectories, and time-resolved fluorescence spectra. See DOI: https://doi.org/10.1039/d6sc01160h. An electronic repository containing example input geometry optimizations and files to initiate trajectories using L-PDFT is available at: https://github.com/helenclifford/LPDFT_HBQ_Dynamics.^104^
