# Supplementary material for: Ultrafast excited-state proton transfer dynamics using linearized pair-density functional theory
Source: Chem Sci. 2026 Jun 22. Online ahead of print. doi: 10.1039/d6sc01160h (PMC13338854; doi:10.1039/d6sc01160h)
Supplement: SC-OLF-D6SC01160H-s001 [file SC-OLF-D6SC01160H-s001.pdf]

Supplementary Information (SI) for Chemical Science.
This journal is © The Royal Society of Chemistry 2026

SUPPLEMENTARY INFORMATION: Ultrafast Excited State Proton Transfer Dynamics Using Linearized Pair-Density Functional Theory

Helen S. Clifford,[†] Aniruddha Seal,[†] and Laura Gagliardi[*,†,‡]

†*Department of Chemistry and Chicago Center for Theoretical Chemistry, University of Chicago, Chicago, IL 60637, USA*

‡*Pritzker School of Molecular Engineering, University of Chicago, Chicago, IL 60637, USA*

E-mail: lgagliardi@uchicago.edu

(Dated: June 29, 2026)

Contents

SI Methods **3**

SII Active Space Stability for L-PDFT Dynamics **4**

SIII Selection of Initial Conditions for ESIPT **6**

SIV Supplementary Figures **8**

SV Analysis of SA-CASSCF Wavefunctions **8**

SVI L-PDFT Potential Energy Surfaces **11**

SVII Energy Conservation of Trajectories **12**

SVIII Simulated Time-Resolved Fluorescence **14**

References **20**

SI Methods

All calculations, performed in this study utilized `PySCF-Forge`[S1](commit `ffb58e6`), which is an extension module for the `PySCF`[S2,S3] software package (version 2.9.0). All calculations used `csf_solver` and all geometry optimizations used the `geomeTRIC` optimizer package[S4] (version 1.0), both implemented in `PySCF`. Additionally, all calculations used the 6-31G** basis set,[S5–S7] the tPBE on-top functional[S8,S9] was utilized for all L-PDFT and SA-CASSCF calculations, and state-averaging was done over the two lowest singlet states (S_0 and S_1). To validate the choice of basis set, the potential energy profiles along the proton-transfer coordinate was computed using the def2-TZVP basis (Section SVI). A (4e,4o) active space was used; the corresponding active-space orbitals for the enol and keto forms of 10-hydroxybenzo[*h*]quinoline (HBQ) are shown in Figure S1. A numerical quadrature grid size of 4 was used for all calculations except for the vertical excitation calculations, PES generation, and all geometry optimizations where a quadrature grid size of 6 was used. Moreover, no spatial symmetry was used in any calculation.

Ab-initio molecular dynamics simulations with L-PDFT were done using `ASE`[S10] (version 3.23.0b1, commit `28a0a1f1988e3`), using the velocity verlet integrator with a timestep of 0.5 fs. Density fitting was utilized to speed up the L-PDFT calculations.[S11] The initial velocities were sampled from a Maxwell-Boltzmann distribution of 300 K.

The generation of initial structures that image the minimum energy pathway of the proton transfer reaction between the enol and keto form of HBQ was performed using the nudged elastic band (NEB)[S12] procedure in `ORCA` (version 5.0)[S13] at the semiempirical PM3 level.[S14] The NEB calculation produced a total of 27 geometries along the proton transfer reaction pathway. Subsequently, the generated geometries were then used in a constrained geometry optimization at the L-PDFT level of theory for both S_0 and S_1. Here, the geometry optimization constraints restricted the O−H and N−H bonds. The S_0 optimized geometries were used to construct the S_0 curve and the S_1 optimized geometries were used to construct the S_1 curve of the PES (Fig. 1b)

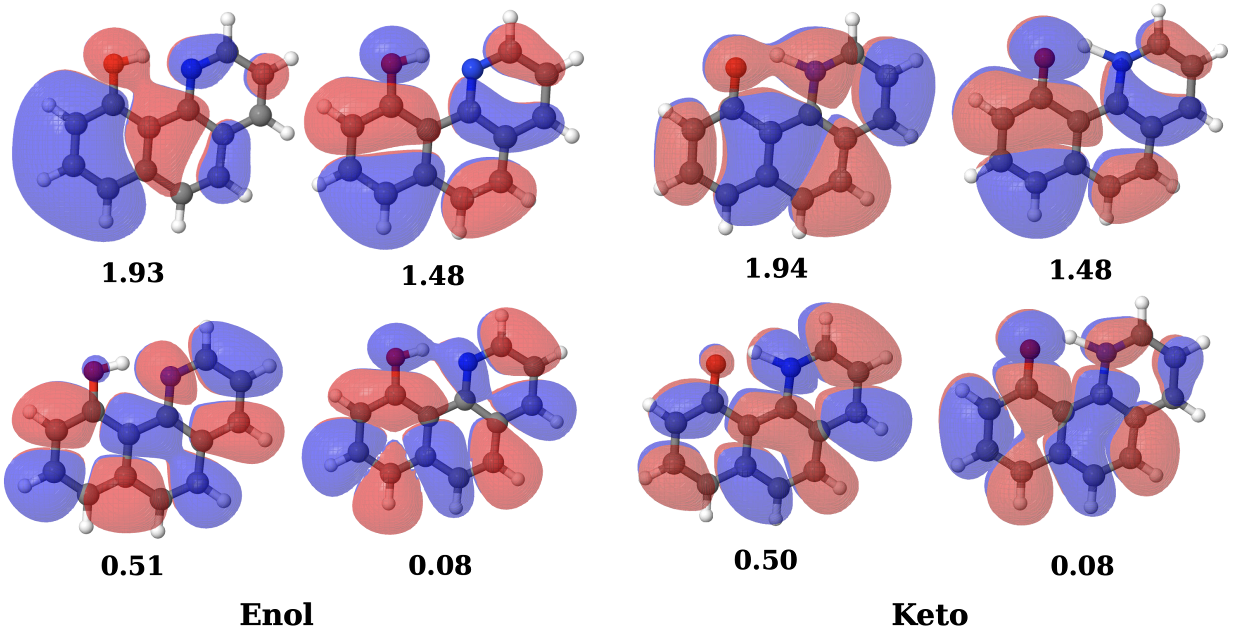

Figure S1: Active space orbitals and natural occupation numbers of HBQ in the enol and keto form. Visualizations are conducted using Jmol[?]

SII Active Space Stability for L-PDFT Dynamics

To confirm the flexibility of (4e, 4o) active space used for the proton transfer reaction, we test its ability to consistently describe both the enol and keto tautomers. We perform a NVE molecular dynamics simulation for both S_0 and S_1 starting from the keto and enol structures respectively. As is shown in Figure S2, the trajectory initiated from the keto structure (ground-state maxima structure) with 1000 timesteps and S_0 L-PDFT nuclear gradients gives way for reforming the enol tautomer. In Figure S3, the trajectory initiated from the enol structure (excited-state maxima structure) over 1000 steps and S_1 L-PDFT nuclear gradients leads to the formation of keto tautomer. This is evident when observing the O−H and N−H distances as a function of time. For both S_0 and S_1, the total energy is stable with no unphysical discontinuities, demonstrating that the active space remains consistent and sufficiently flexible to describe geometries along the reaction pathway for both states.

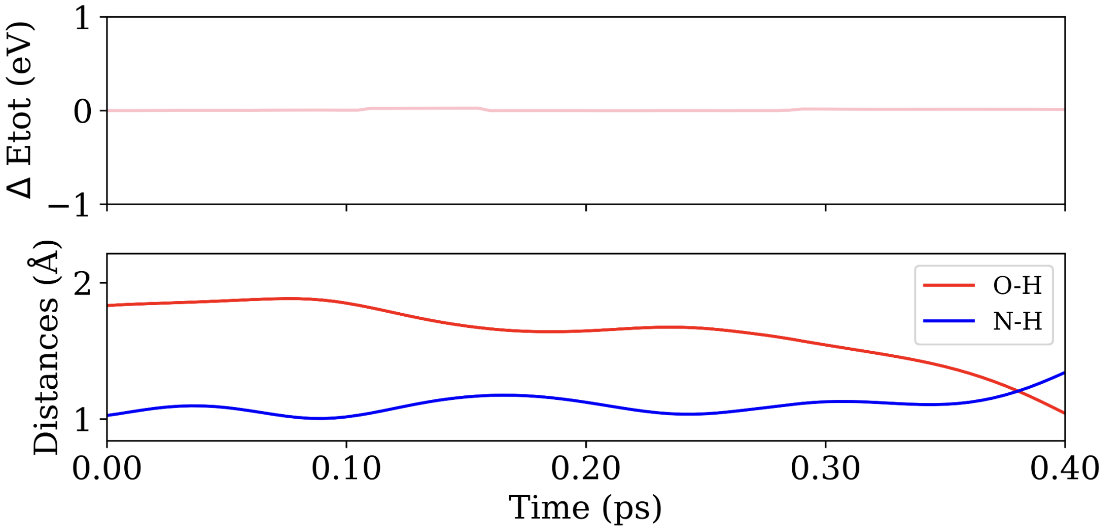

Figure S2: Ground-state relative change in total energy along NVE trajectory initiated from the keto tautomer. Trajectories were propagated with SA(2)-L–tPBE(4e,4o)/6-31G**.

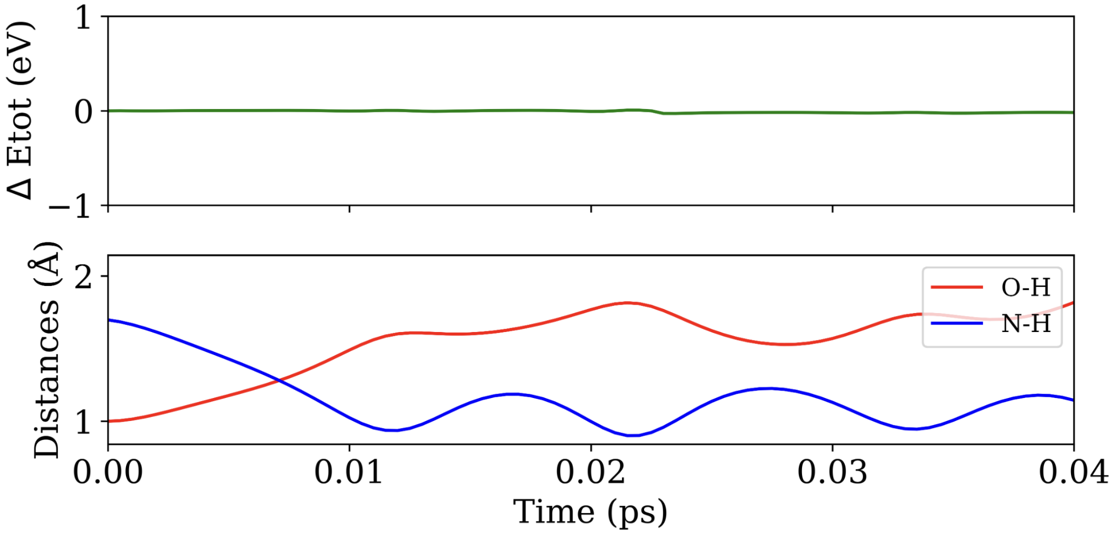

Figure S3: Excited-state relative change in total energy along NVE trajectory initiated from the enol tautomer. Trajectories were propagated with SA(2)-L–tPBE(4e,4o)/6-31G**.

SIII Selection of Initial Conditions for ESIPT

A representative schematic of how the initial conditions for the ESIPT trajectories were chosen are shown in Figure S5. We started with a 1 ps equilibrium sampling of the ground-state enol tautomer basin. Next, we selected 50 configurations from the 1ps equilibrium sampling by selecting a configuration every 20. These 50 configurations were each used in initiating 100 fs simulations on the ground-state surface. Two trajectories were omitted due to their total energies not being conserved (within 1 eV) during the 100 fs simulation. Thus, this results a total of approximately 10,000 geometries. Of these generated configurations, 100 representative, diverse, and unique configurations were chosen for excited-state dynamics.

These 100 configurations were chosen based on Smooth Overlap of Atomic Positions (SOAP) descriptors.[?] SOAP descriptors were computed using a local environment cutoff of $r_{\mathrm{cut}} = 4.5$ Å, with a radial basis expansion truncated at $n_{\mathrm{max}} = 6$ and an angular expansion truncated at $l_{\mathrm{max}} = 4$. Neighbor densities were represented using Gaussians of width $\sigma = 0.5$ Å. The resulting SOAP feature vectors were clustered using mini-batch k-means with `n_clusters` = 100 and `batch_size` = 200, and one configuration from each cluster (closest to the centroid) was selected to yield a set of 100 representative initial conditions. These 100 configurations were propagated on the excited state to simulate the photodynamics of HBQ's proton transfer. Figure S4 shows the selected structures highlighted in the principal-component projection of the SOAP descriptors.

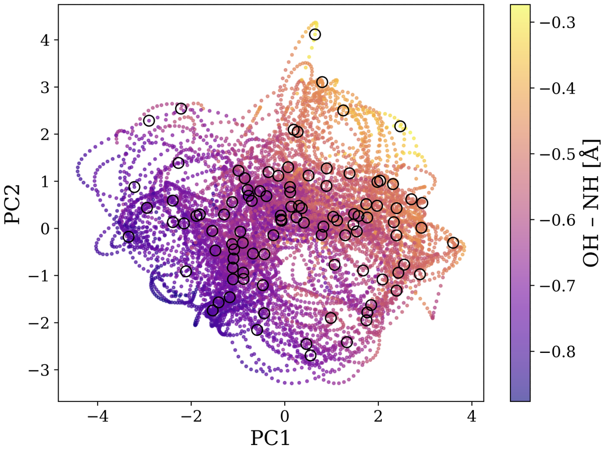

Figure S4: Principal-component projection of the SOAP descriptor space used to quantify structural diversity among Franck–Condon configurations and to select initial conditions for S_1 dynamics. Each point corresponds to a molecular geometry embedded in the reduced SOAP feature space; the 100 representative geometries chosen via k-means clustering to span this space are highlighted as open black circles.

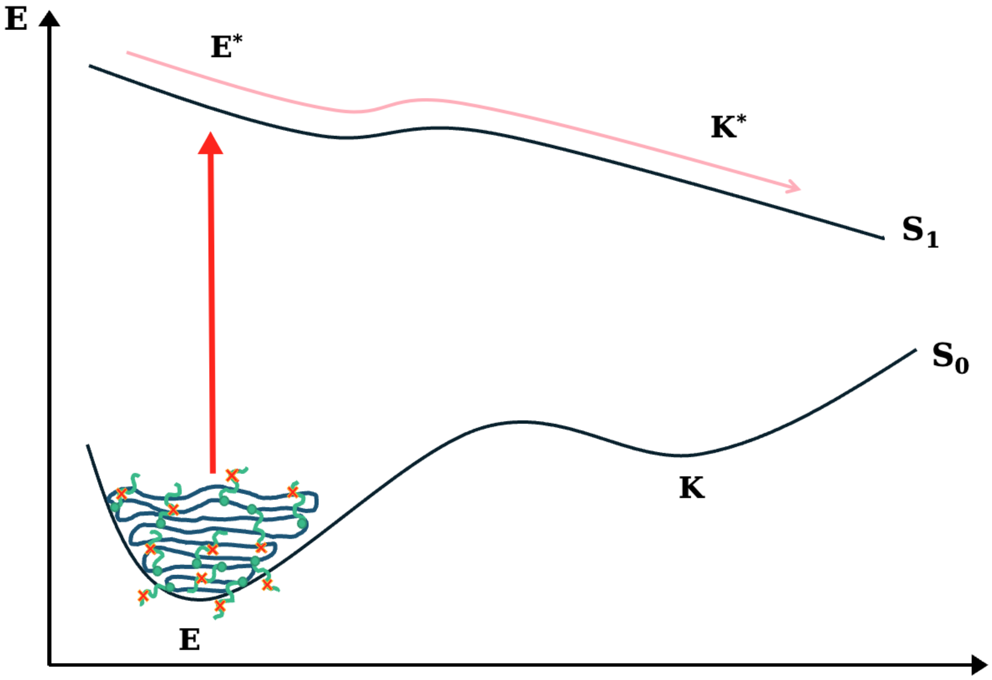

Figure S5: Schematic of HBQ PES depicting the details of the selection of geometries for dynamics simulation of the ESIPT. The dark blue curve shows the S_0 enol tautomer basin. The green circles image the 50 selected configurations from the 1ps equilibrium sampling. The green curved lines represent the 100 fs simulations initiated from the 50 selected configurations. The red Xs are the 100 representative configurations chosen for excited-state dynamics.

SIV Supplementary Figures

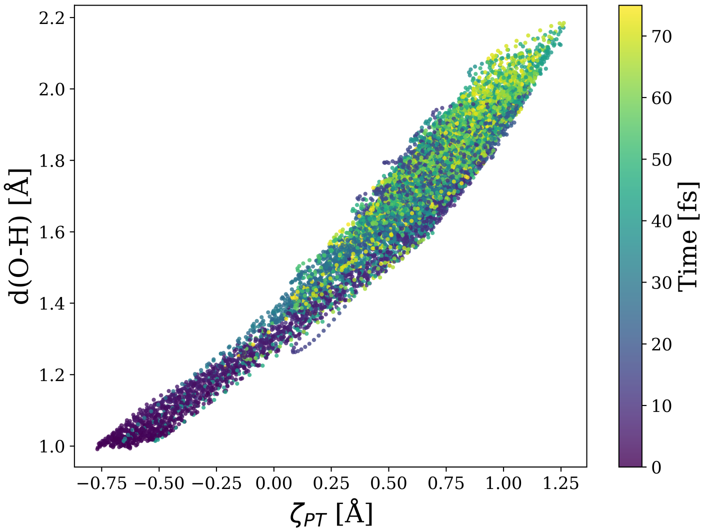

Figure S6: Excited-state evolution of the O−H bond distance for all individual S_1 trajectories as a function of ζ_{PT}. Time is indicated by color through the color bar.

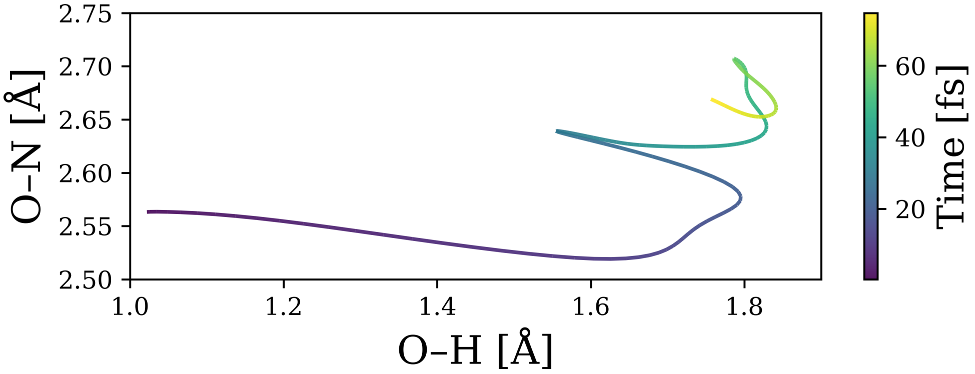

Figure S7: Full average trajectory of the HBQ ESIPT measured by both O−N and O−H distances. Time is indicated by color through the color bar.

SV Analysis of SA-CASSCF Wavefunctions

The M diagnostic is defined as

$$M = \frac{1}{2}\left(2 - n_{\text{HDOMO}} + n_{\text{LUMO}} + \sum_{i \in \text{SOMO}} |n_i - 1|\right) \tag{S1}$$

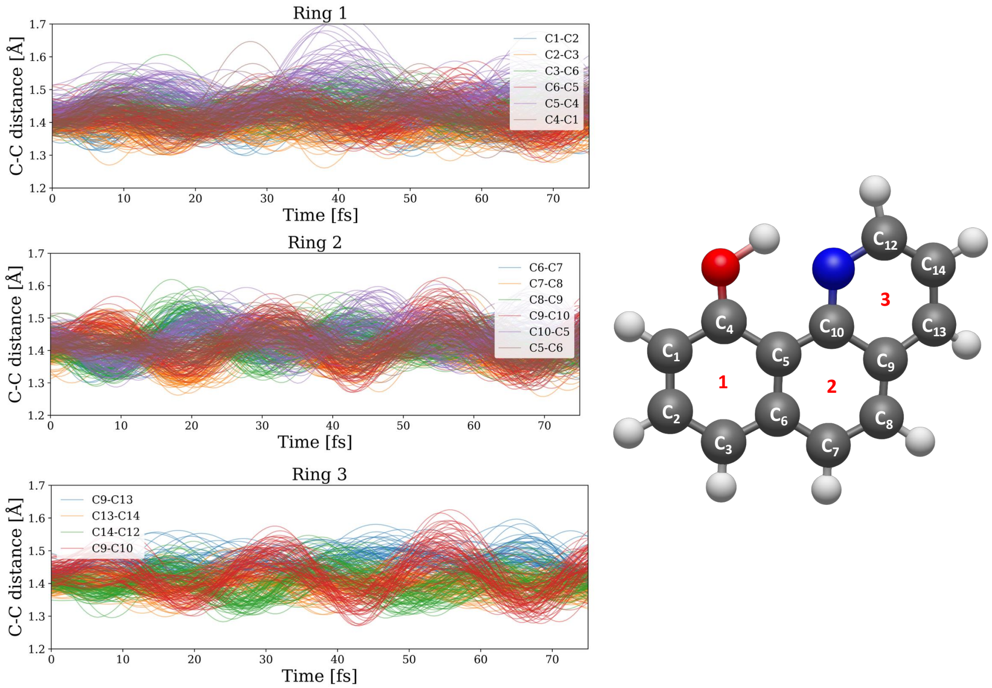

Figure S8: Comparison of C-C distances throughout the molecular backbone of the HBQ molecule as a function of time during the ESIPT process.

where n_{HDOMO}, n_{LUMO} and n_{SOMO} represents the natural orbital occupation number of the highest doubly occupied orbital, the lowest unoccupied orbital, and any singly occupied orbitals within the given active space respectively. A value of $M < 0.05$ represents a primarily single-reference character system and $M > 0.1$ indicates significant multireference character.

The calculation of the M diagnostic along S_1 utilized the S_1 geometries used in generation of the potential energy profile along the proton transfer coordinate (Figure 1). Along the energetically favorable pathway on S_1, we obtain $M \approx 0.37$ (enol), 0.48 (near the transition region), and 0.49 (keto), with the full profile reported in table S1.

Furthermore, we analyze the CI expansion of the excited state SA(2)-CASSCF wave function. The active space consists of four $\pi\pi^*$ orbitals shown in Figure S1. We denote the orbitals as ϕ_1, ϕ_2, ϕ_3, ϕ_4 with natural orbital occupation numbers of 1.93, 1.48, 0.51, and 0.08 at the enol geometry. Across the proton-transfer coordinate, the S1 wave function's composition remains qualitatively consistent. The dominant contributions are:

Table S1: M-diagnostic values of HBQ for S_1 across the ESIPT reaction coordinate.

Geometry Index	M (S_1)
1	0.37
2	0.36
3	0.37
4	0.36
5	0.37
6	0.37
7	0.37
8	0.36
9	0.37
10	0.33
11	0.36
12	0.41
13	0.48
14	0.48
15	0.49
16	0.5
17	0.5
18	0.51
19	0.51
20	0.54
21	0.57
22	0.53
23	0.5
24	0.49
25	0.49
26	0.49
27	0.49
Average M	0.44

- $\pi_2 \rightarrow \pi_3^*$ with 30% weight: Singly excited CSF from π_2 to the lower antibonding π^*
- $\pi_2 \rightarrow \pi_4^*$ with 18–19% weight: Singly excited CSF from π_2 into the higher π^*
- $\pi_1 \rightarrow \pi_3^*$ with 15% weight.: Singly excited CSF from the first bonding π into π_3^*
- $\pi_1\pi_2 \rightarrow \pi_3^*\pi_4^*$ with 14–15% weight: Doubly excited CSF

The S_1 state has predominantly $\pi\pi^*$ character, as also noted previously[S15] with no single dominant configuration reflected in the M diagnostic reported. The wave function character

is stable across the geometries examined here, with only small variations in CSF weights. The CI vector is the expansion of the excited state wave function as a sum over slater determinants. The coefficient is representative of how much the determinant contributes to the overall wave function. The weight is the coefficient squared in percent. Shown below is the breakdown of the the ground-state enol structure's and excited-state keto structure's electronic structure in terms of the CI coefficients and determinants.

Table S2: Analysis of CI coefficients and Slater determinants of the ground-state enol minima

**Coefficient**	**Weight**	**Determinant**
+0.527711	27.8479%	$\vert\phi_1(2)\ \phi_2(2)\ \phi_3(0)\ \phi_4(0)>$
-0.379356	14.3911%	$\vert\phi_1(\uparrow)\ \phi_2(2)\ \phi_3(\downarrow)\ \phi_4(0)>$
-0.379356	14.3911%	$\vert\phi_1(\downarrow)\ \phi_2(2)\ \phi_3(\uparrow)\ \phi_4(0)>$
+0.309591	9.5846%	$\vert\phi_1(2)\ \phi_2(\uparrow)\ \phi_3(0)\ \phi_4(\downarrow)>$
+0.309591	9.5846%	$\vert\phi_1(2)\ \phi_2(\downarrow)\ \phi_3(0)\ \phi_4(\uparrow)>$
+0.209279	4.3798%	$\vert\phi_1(0)\ \phi_2(2)\ \phi_3(2)\ \phi_4(0)>$
+0.176690	3.1219%	$\vert\phi_1(\downarrow)\ \phi_2(\downarrow)\ \phi_3(\uparrow)\ \phi_4(\uparrow)>$
+0.176690	3.1219%	$\vert\phi_1(\uparrow)\ \phi_2(\uparrow)\ \phi_3(\downarrow)\ \phi_4(\downarrow)>$
-0.172010	2.9588%	$\vert\phi_1(\downarrow)\ \phi_2(\uparrow)\ \phi_3(\uparrow)\ \phi_4(\downarrow)>$
-0.172010	2.9588%	$\vert\phi_1(\uparrow)\ \phi_2(\downarrow)\ \phi_3(\downarrow)\ \phi_4(\uparrow)>$
+0.132812	1.7639%	$\vert\phi_1(2)\ \phi_2(0)\ \phi_3(0)\ \phi_4(2)>$
-0.092377	0.8534%	$\vert\phi_1(\uparrow)\ \phi_2(2)\ \phi_3(0)\ \phi_4(\downarrow)>$
-0.092377	0.8534%	$\vert\phi_1(\downarrow)\ \phi_2(2)\ \phi_3(0)\ \phi_4(\uparrow)>$
-0.086133	0.7419%	$\vert\phi_1(0)\ \phi_2(2)\ \phi_3(0)\ \phi_4(2)>$
-0.075848	0.5753%	$\vert\phi_1(0)\ \phi_2(\uparrow)\ \phi_3(2)\ \phi_4(\downarrow)>$
-0.075848	0.5753%	$\vert\phi_1(0)\ \phi_2(\downarrow)\ \phi_3(2)\ \phi_4(\uparrow)>$
-0.073861	0.5455%	$\vert\phi_1(2)\ \phi_2(0)\ \phi_3(2)\ \phi_4(0)>$
+0.067174	0.4512%	$\vert\phi_1(2)\ \phi_2(\uparrow)\ \phi_3(\downarrow)\ \phi_4(0)>$
+0.067174	0.4512%	$\vert\phi_1(2)\ \phi_2(\downarrow)\ \phi_3(\uparrow)\ \phi_4(0)>$

SVI L-PDFT Potential Energy Surfaces

Figure S9a and Figure S9b depict ground-state (S_0) and first excited singlet-state (S_1) potential-energy profiles along the ESIPT coordinate(ζ_{PT}) computed with ground and excited-state optimized geometries respectively. The energy profiles are computed with SA(2)-CAS(4,4) and L-PDFT/tPBE using the 6-31G** basis set. Energies are referenced to the S_0

Table S3: Analysis of CI coefficients and Slater determinants of the excited-state keto minima

**Coefficient**	**Weight**	**Determinant**
+0.388220	15.0715%	$\| \phi_1(2)\ \phi_2(\uparrow)\ \phi_3(\downarrow)\ \phi_4(0)>$
-0.388220	15.0715%	$\|\phi_1(2)\ \phi_2(\downarrow)\ \phi_3(\uparrow)\ \phi_4(0)\ >$
-0.306672	9.4048%	$\|\phi_1(2)\ \phi_2(\uparrow)\ \phi_3(0)\ \phi_4(\downarrow)>$
+0.306672	9.4048%	$\|\phi_1(2)\ \phi_2(\downarrow)\ \phi_3(0)\ \phi_4(\uparrow)>$
-0.277758	7.7149%	$\|\phi_1(\downarrow)\ \phi_2(\uparrow)\ \phi_3(2)\ \phi_4(0)>$
+0.277758	7.7149%	$\|\phi_1(\uparrow)\ \phi_2(\downarrow)\ \phi_3(2)\ \phi_4(0)>$
-0.273320	7.4704%	$\|\phi_1(\uparrow)\ \phi_2(\uparrow)\ \phi_3(\downarrow)\ \phi_4(\downarrow)>$
+0.273320	7.4704%	$\|\phi_1(\downarrow)\ \phi_2(\downarrow)\ \phi_3(\uparrow)\ \phi_4(\uparrow)>$
+0.172678	2.9818%	$\|\phi_1(2)\ \phi_2(0)\ \phi_3(\downarrow)\ \phi_4(\uparrow)>$
-0.172678	2.9818%	$\|\phi_1(2)\ \phi_2(0)\ \phi_3(\uparrow)\ \phi_4(\downarrow)>$
+0.167215	2.7961%	$\|\phi_1(\downarrow)\ \phi_2(\uparrow)\ \phi_3(\uparrow)\ \phi_4(\downarrow)>$
-0.167215	2.7961%	$\|\phi_1(\uparrow)\ \phi_2(\downarrow)\ \phi_3(\downarrow)\ \phi_4(\uparrow)>$
+0.120033	1.4408%	$\|\phi_1(\downarrow)\ \phi_2(2)\ \phi_3(\uparrow)\ \phi_4(0)>$
-0.120033	1.4408%	$\|\phi_1(\uparrow)\ \phi_2(2)\ \phi_3(\downarrow)\ \phi_4(0)>$
+0.092169	0.8495%	$\|\phi_1(\downarrow)\ \phi_2(0)\ \phi_3(2)\ \phi_4(\uparrow)>$
-0.092169	0.8495%	$\|\phi_1(\uparrow)\ \phi_2(0)\ \phi_3(2)\ \phi_4(\downarrow)>$
+0.088337	0.7803%	$\|\phi_1(0)\ \phi_2(\uparrow)\ \phi_3(2)\ \phi_4(\downarrow)>$
-0.088337	0.7803%	$\|\phi_1(0)\ \phi_2(\downarrow)\ \phi_3(2)\ \phi_4(\uparrow)>$
+0.086085	0.7411%	$\|\phi_1(\downarrow)\ \phi_2(\uparrow)\ \phi_3(0)\ \phi_4(2)>$
-0.086085	0.7411%	$\|\phi_1(\uparrow)\ \phi_2(\downarrow)\ \phi_3(0)\ \phi_4(2)>$
-0.071541	0.5118%	$\|\phi_1(\uparrow)\ \phi_2(0)\ \phi_3(\downarrow)\ \phi_4(2)>$
+0.071541	0.5118%	$\|\phi_1(\downarrow)\ \phi_2(0)\ \phi_3(\uparrow)\ \phi_4(2)>$

minimum for each method. Figure S9c depicts the potential-energy profiles along the ESIPT coordinate(ζ_{PT}) using the def2-TZVP basis set with SA(2)-CAS(4,4) and L-PDFT/tPBE utilizing ground and excited-state optimized geometries for the ground and excited-state respectively. Figure S9d shows the potential-energy profiles along the ESIPT coordinate(ζ_{PT}) using the 6-31G** basis set with SA(3)-CAS(4,4) and L-PDFT/tPBE.

SVII Energy Conservation of Trajectories

Trajectories were classified as non-energy-conserving and excluded from the analysis if their maximum total-energy drift exceeded 1 eV relative to the initial energy after the first 0.05 ps of propagation. Applying this objective criterion resulted in the retention of 72 of the original 100 trajectories. To assess the sensitivity of our results to this filtering procedure, we

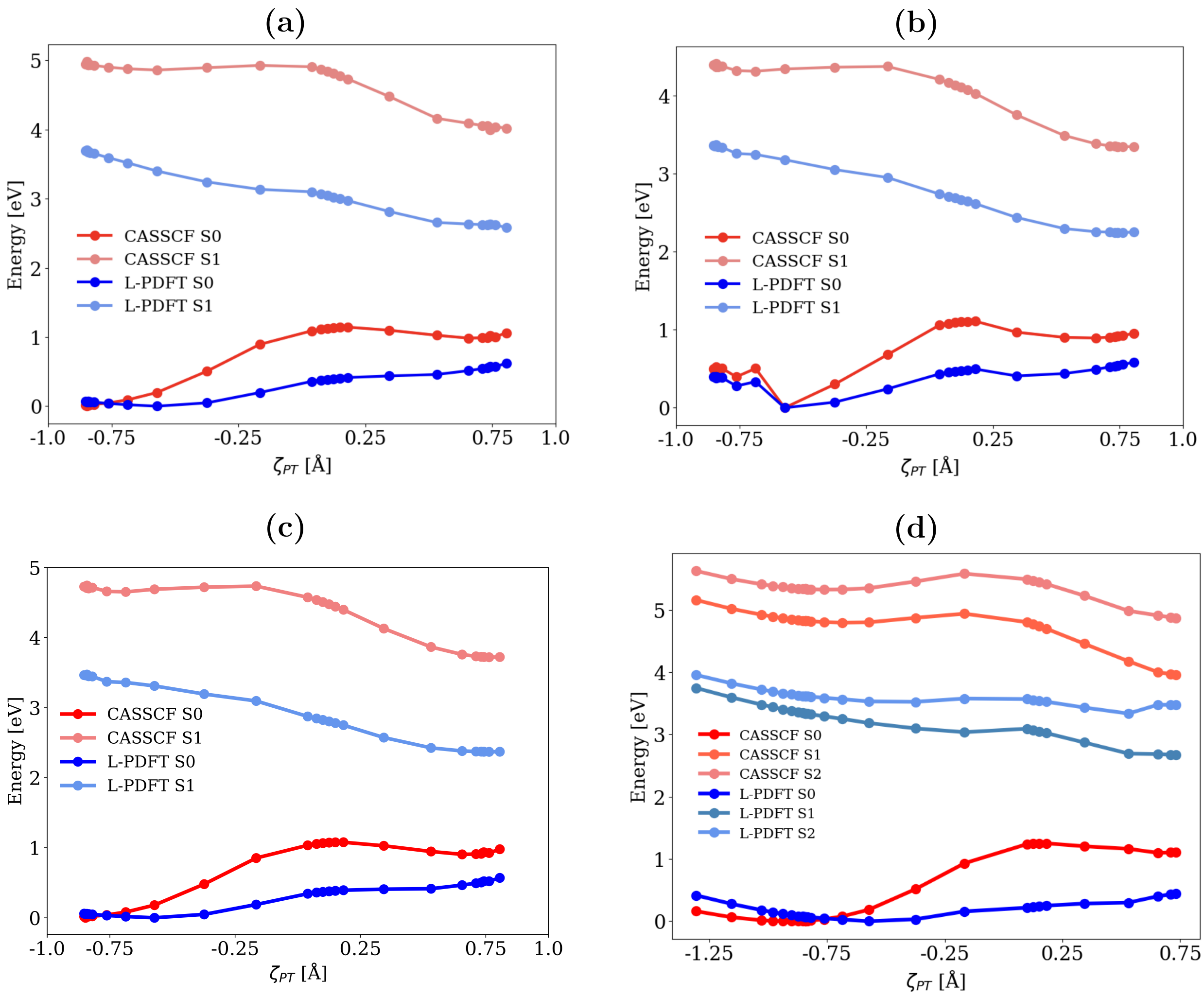

Figure S9: Potential energy surfaces of the ESIPT of HBQ as a function of ζ_{PT}. (a) L-PDFT and SA-CASSCF potential energy surfaces using geometries optimized at the S_0 level of theory. (b) using geometries optimized at the S_1 level of theory. (c) computed using the def2-TZVP basis set. (d) SA(3)-CAS(4,4) and L-PDFT/tPBE potential energy surfaces using the 6-31G** basis set, with energies referenced to the S_0 minimum for each method.

repeated the analysis using all 100 trajectories. The resulting ESIPT timescale was 15.956 ± 7.500 fs, compared to 16.017 ± 8.262 fs (reported as 16 ± 8 fs) obtained using the filtered set of 72 trajectories. The values are well within the statistical uncertainty. Inclusion of all trajectories does not alter any of the qualitative conclusions. In both cases, the predicted proton-transfer timescale remains ultrafast and consistent with previous experimental and theoretical studies of HBQ. These results demonstrate that the reported ESIPT timescale and mechanistic conclusions are robust with respect to the trajectory-filtering procedure.

As is evident, the ESIPT timescale calculated with all 100 trajectories is nearly equivalent to the timescale calculated with the discarded trajectories. In addition, including these trajectories does not change our qualitative interpretations and conclusions of our L-PDFT study, as both timescales reflect an ultrafast timescale. Thus, we take our results from discarding the trajectories exceeding 1 eV in total energy drift as being accurate and representative of our L-PDFT dynamics.

Shown in Figure S10, it is shown that when excluding vs. including trajectories marked as not conserving of total energy, the qualitative results and conclusions that could be made remain the same. The primary result remains, from Figure S10a and Figure S10b, that the formation of the keto carbonyl takes place within our estimated L-PDFT ESIPT timescale (16 fs). Our conclusion from Figure S10c and Figure S10d remains that the O–N distance stays nearly constant over the course of the predicted ESIPT timescale.

Additionally, in Figure S11 and Figure S12, no significant changes are evident from comparing the original manuscript trajectories vs the figures with excluded trajectories.

SVIII Simulated Time-Resolved Fluorescence

To provide a closer comparison with the experimental time-resolved fluorescence (TRF) measurements of Lee *et al.*,[S16] we simulated the TRF decay of the *enol* emission from the L-PDFT trajectories following the procedure described by Barbatti and co-workers.[S17,S18] The experimental TRF signal monitors the decay of the *enol* emission, which is directly associated with depletion of the initially excited enol population during ESIPT. In our analysis, the *enol* region is defined by $\zeta_{\mathrm{PT}} < -0.15\ \mathring{A}$ where $\zeta_{\mathrm{PT}} = \mathrm{d(O-H)} - \mathrm{d(N-H)}$.

The spontaneous emission rate for the $S_1 \rightarrow S_0$ transition is written as

$$\Gamma_{1\rightarrow 0}(t) = \frac{2\alpha}{m_e \hbar c^2} f_{1\rightarrow 0}(t) \Delta E(t)^2, \tag{S2}$$

where α is the fine-structure constant, m_e is the electron mass, $\hbar$ is the reduced Planck

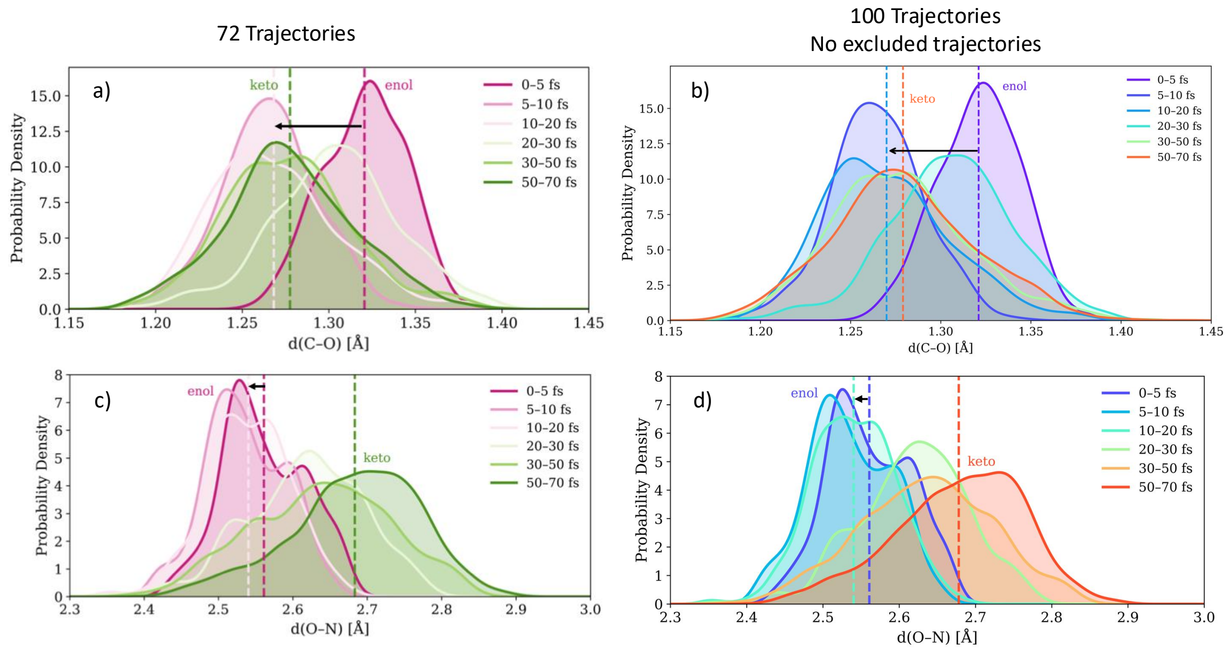

Figure S10: Comparison between excited-state structural evolution along the S_1 trajectories for 72 trajectories vs 100 trajectories: a) Time-resolved probability density of the C–O bond length. Kernel density estimates (KDEs) of $d(\mathrm{C-O})$ are shown for successive time blocks (0–5, 5–10, 10–20, 20–30, 30–50, and 50–70 fs), with each curve normalized to unit area. Curves are colored by time-block order. Dashed vertical lines mark the mean $d(\mathrm{C-O})$ in the time blocks 0–5 fs which marks the "enol" form, 10–20 fs marks the "nascent keto form" and the final 50–70 fs block marks the "relaxed keto form" of HBQ. b) Same as panel a) but computed without excluding any trajectories due to total energy drift. c) Same as panel a), but for the O–N distance. d) Same as panel c), but computed without excluding any trajectories due to total energy drift. The respective black arrows on each of the plots highlight the net shift in the distribution over the average reaction time of 16 fs.

constant, c is the speed of light, $f_{1\to 0}(t)$ is the oscillator strength, and $\Delta E(t)$ is the vertical $S_1 \to S_0$ energy (see Figure S15). In atomic units, this expression becomes

$$\Gamma_{1\to 0}(t) = \frac{4\alpha}{3} f_{1\to 0}(t)\Delta E(t)^2. \tag{S3}$$

Because analytic transition dipole moments are not yet available for L-PDFT, we evaluate the simulated TRF using two limiting approximations. First, we assume a constant oscillator

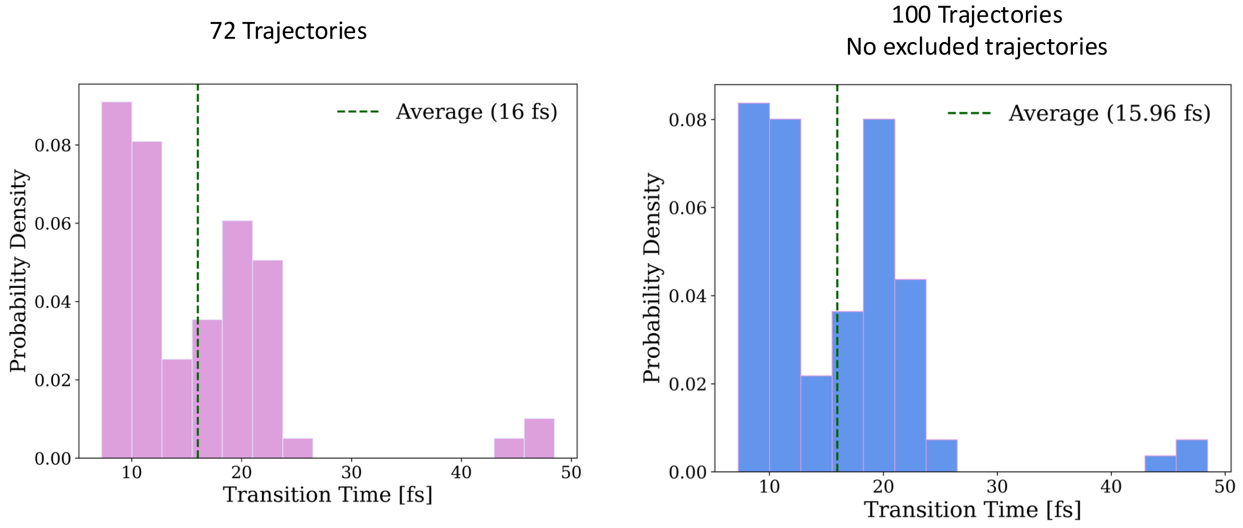

Figure S11: Comparison of the distribution of all 72 trajectory transition times (defined as the time of first occurance of $\zeta_{\mathrm{PT}} = 0.79$ Å). The left is the plot of all total energy conserving trajectories. Right is the plot of all 100 trajectories. The vertical green marker denotes the average time each trajectory is deemed complete.

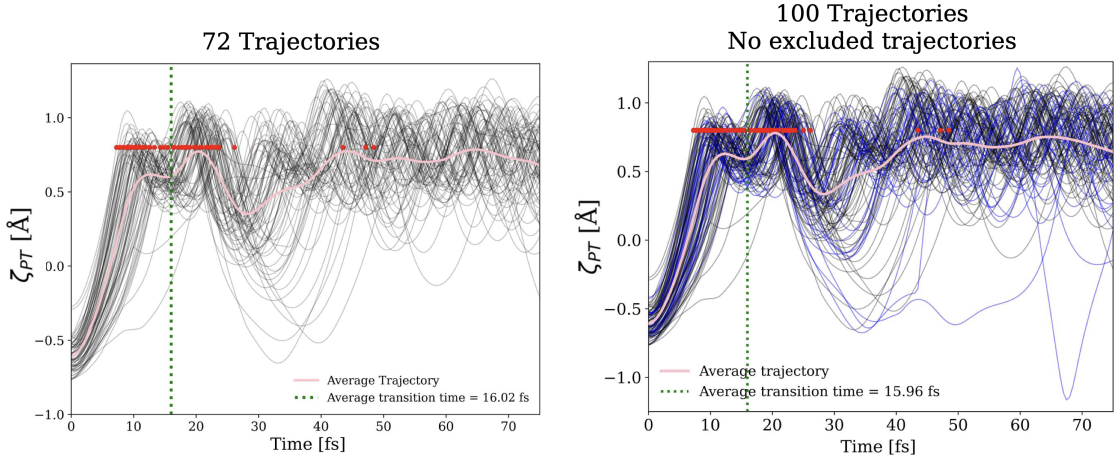

Figure S12: Comparison of the time evolution of the ESIPT in HBQ along the proton-transfer coordinate ζ_{PT} on the S_1 surface. The left is the plot of all total energy conserving trajectories. Right is the plot of all 100 trajectories. The ensemble-averaged trajectory is shown in pink. The green vertical line marks the mean completion time, defined as the first passage into the keto basin. The red dots represent the point at which each individual trajectory achieved the defined transition time. The blue trajectory lines represent the trajectories that were originally excluded due to lack of total energy conservation.

strength, $f_{1\to 0} = 1$, throughout the ESIPT dynamics. Under this approximation,

$$\Gamma^{(f)}(t) = \frac{4\alpha}{3}\Delta E(t)^2. \tag{S4}$$

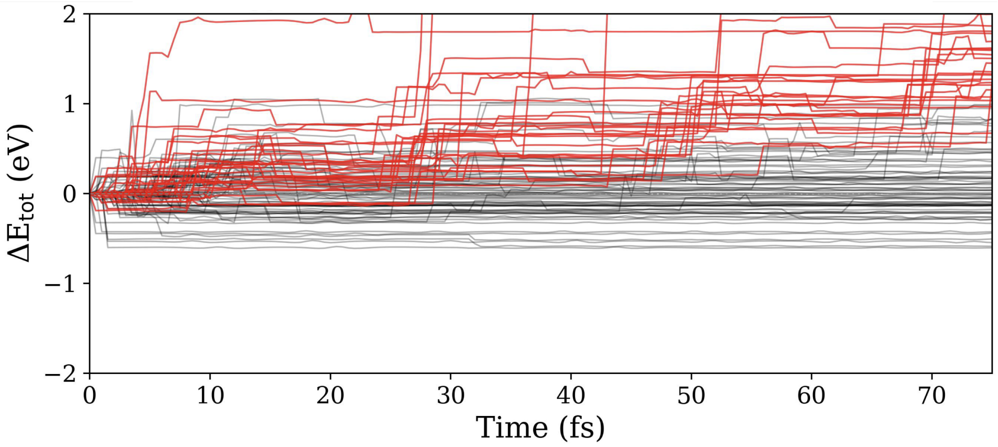

Figure S13: Total energy conservation across 100 trajectories. Black lines correspond to energy conserving and red lines (28) correspond to non-energy conserving trajectories.

Second, we assume a constant transition dipole moment, $|\boldsymbol{\mu}_{1\to0}| = 1$ a.u. The oscillator strength and transition dipole moment are related in atomic units by

$$f_{1\to0}(t) = \frac{2}{3}\Delta E(t)|\boldsymbol{\mu}_{1\to0}|^2. \tag{S5}$$

Substituting this relation into eq. (S3) and taking $|\boldsymbol{\mu}_{1\to0}| = 1$ a.u. gives,

$$\Gamma^{(\mu)}(t) = \frac{4}{3}\Delta E(t)^3. \tag{S6}$$

The simulated ensemble-averaged fluorescence intensity is then obtained by averaging the trajectory-resolved emission rates over the $N = 72$ energy-conserving L-PDFT trajectories,

$$I^{(x)}(t) = \frac{1}{N}\sum_{i=1}^{N}\Gamma_i^{(x)}(t), \qquad x \in \{f, \mu\}. \tag{S7}$$

The standard error of the mean fluorescence intensity is computed as

$$SE^{(x)}(t) = \frac{1}{\sqrt{N}} \sqrt{\frac{1}{N} \sum_{i=1}^{N} \left[\Gamma_i^{(x)}(t) - I^{(x)}(t) \right]^2}. \tag{S8}$$

Here, the excited-state population is assumed to remain unity over the analyzed time window, consistent with the absence of nonadiabatic decay in the present adiabatic L-PDFT trajectories. Thus, the simulated TRF reflects the decay of the enol-emission signal due to ESIPT-induced changes in the emitting ensemble rather than population loss from S_1.

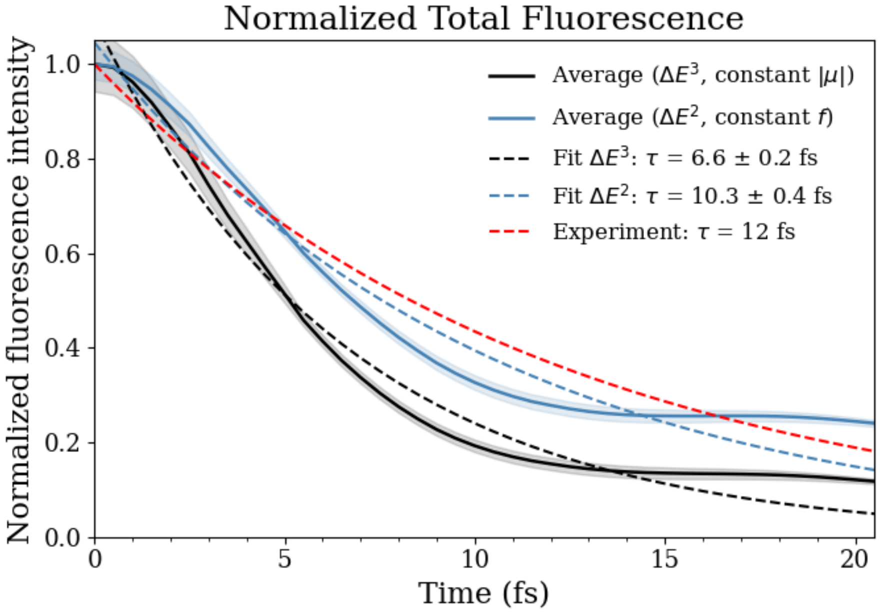

Figure S14: Simulated time-resolved fluorescence intensity of HBQ during ESIPT in the first excited singlet state. Intensity normalized to its value at t = 0. Black solid line is the trajectory average assuming a constant transition dipole moment $|\mu| = 1$ for $\Gamma \propto \Delta E^3$. Blue solid line is the trajectory average assuming a constant oscillator strength $f = 1$ for $\Gamma \propto \Delta E^2$. Grey-shaded and blue-shaded regions represent the standard errors given by eq. (S8) for the constant transition dipole moment ensemble average and constant oscillator strength ensemble average. Dashed black and blue lines are monoexponential fits $A_1 e^{-\frac{t}{\tau}}$ to the respective averages. Red dashed line is a monoexponential decay with $\tau = 12$ fs representing the experimental time-resolved fluorescence timescale found by Lee *et al.*

The simulated TRF traces are normalized and fit to a single-exponential decay (Figure S14). Using the constant oscillator-strength approximation, $f_{1\to0} = 1$, we obtain a decay time constant of 10.3 fs. Using the constant transition-dipole approximation, $|\boldsymbol{\mu}_{1\to0}| = 1$ a.u.,

we obtain a decay time constant of 6.6 fs. Overall, both approximate weighting schemes reproduce an ultrafast decay of the enol-emission signal, consistent with the experimental enol-emission TRF decay time of 12 fs. The remaining difference from experiment may arise, in part, from the use of constant oscillator strengths or transition dipole moments. Analytic transition dipole moments for L-PDFT, currently under development, will enable direct computation of the time-dependent radiative weights along the trajectories and should provide a more rigorous route to quantitative agreement with experimental TRF spectra.

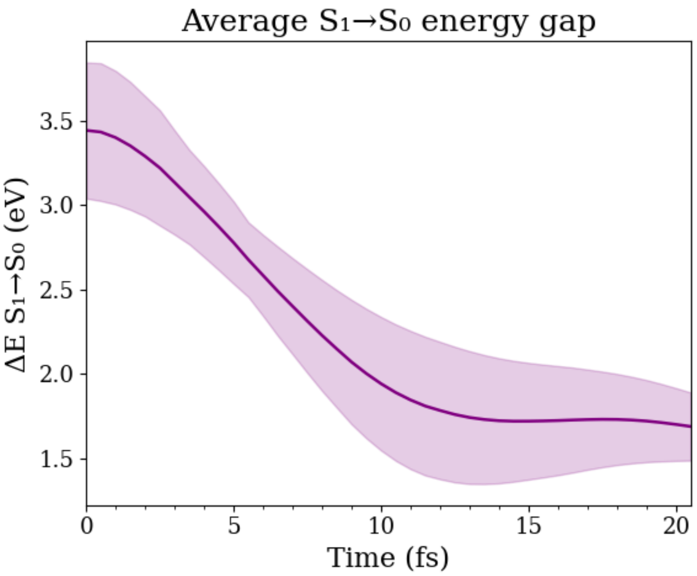

Figure S15: Energy gap between first excited singlet and ground state of HBQ over the course of the ESIPT process. The dark purple line represents the average energy gap for all trajectories. Shaded purple region represents the standard error across all trajectories.

References

[S1] *PySCF-Forge.* Github, 2025. `https://github.com/pyscf/pyscf-forge`, Available via the Internet. Accessed 28 Aug. 2025.

[S2] Sun, Q.; Berkelbach, T. C.; Blunt, N. S.; Booth, G. H.; Guo, S.; Li, Z.; Liu, J.; McClain, J. D.; Sayfutyarova, E. R.; Sharma, S.; Wouters, S.; Chan, G. K. L. PySCF: The Python-based simulations of chemistry framework. *WIREs Comput. Mol. Sci.* **2018**, *8*, no. e1340.

[S3] Sun, Q.; Zhang, X.; Banerjee, S.; Bao, P.; Barbry, M.; Blunt, N. S.; Bogdanov, N. A.; Booth, G. H.; Chen, J.; Cui, Z.-H.; et al. Recent developments in the PySCF program package. *J. Chem. Phys.* **2020**, *153*, no. 024109.

[S4] Wang, L.-P.; Song, C. Geometry Optimization Made Simple With Translation and Rotation Coordinates. *J. Chem. Phys.* **2016**, *144*, no. 214108.

[S5] Hehre, W. J.; Ditchfield, R.; Pople, J. A. Self-Consistent Molecular Orbital Methods. XII. Further Extensions of Gaussian-Type Basis Sets for Use in Molecular Orbital Studies of Organic Molecules. *J. Chem. Phys.* **1972**, *56*, 2257–2261.

[S6] Ditchfield, R.; Hehre, W. J.; Pople, J. A. Self-Consistent Molecular-Orbital Methods. IX. An Extended Gaussian-Type Basis for Molecular-Orbital Studies of Organic Molecules. *J. Chem. Phys.* **1971**, *54*, 724–728.

[S7] Hariharan, P. C.; Pople, J. A. The influence of polarization functions on molecular orbital hydrogenation energies. *Theoret. Chim. Acta* **1973**, *28*, 213–222.

[S8] Li Manni, G.; Carlson, R. K.; Luo, S.; Ma, D.; Olsen, J.; Truhlar, D. G.; Gagliardi, L. Multiconfiguration Pair-Density Functional Theory. *J. Chem. Theory Compt.* **2014**, *10*, 3669–3680.

[S9] Perdew, P.; Burke, K.; Ernzerhof, M. Generalized Gradient Approximation Made Simple. *Phys. Rev. Lett.* **1996**, *77*, 3865–3868.

[S10] Larsen, A. H. et al. The atomic simulation environment—a Python library for working with atoms. *J. Phys. Condens. Matter* **2017**, *29*, 273002.

[S11] Scott, T. R.; Oakley, M. S.; Hermes, M. R.; Sand, A. M.; Lindh, R.; Truhlar, D. G.; Gagliardi, L. Analytic gradients for multiconfiguration pair-density functional theory with density fitting: Development and application to geometry optimization in the ground and excited states. *J. Chem. Phys.* **2021**, *154*.

[S12] Ásgeirsson, V.; Birgisson, B. O.; Bjornsson, R.; Becker, U.; Neese, F.; Riplinger, C.; Jónsson*, H. Nudged Elastic Band Method for Molecular Reactions Using Energy-Weighted Springs Combined with Eigenvector Following. *J. Chem. Theory Compt.* **2021**, *18*, 4929–4945.

[S13] Neese, F. Software update: The ORCA program system—Version 5.0. *WIRES Comput. Molec. Sci.* **2022**, *12*, e1606.

[S14] Stewart, J. J. P. Optimization of parameters for semiempirical methods I. Method. *J. Comput, Chem.* **1989**, *10*, 209–220.

[S15] Schriever, C.; Barbatti, M.; Stock, K.; Aquino, A. J.; Tunega, D.; Lochbrunner, S.; Riedle, E.; de Vivie-Riedle, R.; Lischka, H. The interplay of skeletal deformations and ultrafast excited-state intramolecular proton transfer: Experimental and theoretical investigation of 10-hydroxybenzo[h]quinoline. *Chem. Phys.* **2008**, *347*, 446–461.

[S16] Lee, J.; Kim, C. H.; Joo, T. Active Role of Proton in Excited State Intramolecular Proton Transfer Reaction. *J. Phys. Chem. A* **2013**, *117*, 1400–1405.

[S17] Sülzner, N.; Mattos, R. S.; Barbatti, M. Molecular Dynamics of the Ultrafast Excited-

State Proton Transfer of the Super-Photoacid NM7HQ+ to the Aprotic Solvent DMSO. *J. Phys. Chem. Lett.* **2025**, *16*, 9124–9134.

[S18] Braun, G.; Itamar Borges, J.; Aquino, A. J. A.; Lischka, H.; Plasser, F.; do Monte, S. A.; Ventura, E.; Mukherjee, S.; Barbatti, M. Non-Kasha fluorescence of pyrene emerges from a dynamic equilibrium between excited states. *J. Chem. Phys.* **2022**, *157*, 154305.
